# Magnitude and Durability of the Antibody Response to mRNA-Based Vaccination Among SARS-CoV-2 Seronegative and Seropositive Health Care Personnel

**DOI:** 10.1093/ofid/ofae009

**Published:** 2024-01-19

**Authors:** Emily J Ciccone, Deanna R Zhu, Annika K Gunderson, Sam Hawke, Rawan Ajeen, Evans K Lodge, Bonnie E Shook-Sa, Haley Abernathy, Haley E Garrett, Elise King, Naseem Alavian, Raquel Reyes, Jasmine L Taylor, Cherese Beatty, Christy Chung, Carmen E Mendoza, David J Weber, Alena J Markmann, Lakshmanane Premkumar, Jonathan J Juliano, Ross M Boyce, Allison E Aiello

**Affiliations:** Division of Infectious Diseases, School of Medicine; Department of Epidemiology, Gillings School of Global Public Health; Department of Epidemiology, Gillings School of Global Public Health; Department of Biostatistics, Gillings School of Global Public Health; Institute for Global Health and Infectious Diseases; Department of Epidemiology, Gillings School of Global Public Health; Department of Biostatistics, Gillings School of Global Public Health; Institute for Global Health and Infectious Diseases; Department of Epidemiology, Gillings School of Global Public Health; Institute for Global Health and Infectious Diseases; Division of Hospital Medicine, School of Medicine, University of North Carolina, Chapel Hill, North Carolina; Division of Hospital Medicine, School of Medicine, University of North Carolina, Chapel Hill, North Carolina; Institute for Global Health and Infectious Diseases; Department of Epidemiology and Robert N. Butler Columbia Aging Center, Mailman School of Public Health, Columbia University, New York, New York; Institute for Global Health and Infectious Diseases; Department of Epidemiology, Gillings School of Global Public Health; Division of Infectious Diseases, School of Medicine; Department of Epidemiology, Gillings School of Global Public Health; Division of Infectious Diseases, School of Medicine; Department of Microbiology and Immunology, School of Medicine, University of North Carolina, Chapel Hill, North Carolina; Division of Infectious Diseases, School of Medicine; Department of Epidemiology, Gillings School of Global Public Health; Division of Infectious Diseases, School of Medicine; Department of Epidemiology, Gillings School of Global Public Health; Department of Epidemiology and Robert N. Butler Columbia Aging Center, Mailman School of Public Health, Columbia University, New York, New York

**Keywords:** antibody response, health care personnel, hybrid immunity, SARS-CoV-2 infection, vaccination

## Abstract

Few studies have described changes in SARS-CoV-2 antibody levels in response to infection and vaccination at frequent intervals and over extended follow-up periods. The purpose of this study was to assess changes in SARS-CoV-2–specific antibody responses among a prospective cohort of health care personnel over 18 months with up to 22 samples per person. Antibody levels and live virus neutralization were measured before and after mRNA-based vaccination with results stratified by (1) SARS-CoV-2 infection status prior to initial vaccination and (2) SARS-CoV-2 infection at any point during follow-up. We found that the antibody response to the first dose was almost 2-fold higher in individuals who were seropositive prior to vaccination, although neutralization titers were more variable. The antibody response induced by vaccination appeared to wane over time but generally persisted for 8 to 9 months, and those who were infected at any point during the study had slightly higher antibody levels over time vs those who remained uninfected. These findings underscore the need to account for SARS-CoV-2 natural infection as a modifier of vaccine responses, and they highlight the importance of frequent testing of longitudinal antibody titers over time. Together, our results provide a clearer understanding of the trajectories of antibody response among vaccinated individuals with and without prior SARS-CoV-2 infection.

Understanding the dynamics of humoral immunity in response to SARS-CoV-2 infection and vaccination is imperative to determine individual- and population-level protection against infection and severe disease. Previous studies have demonstrated that prior SARS-CoV-2 infection “primes” the immune system to respond more robustly to the first mRNA-based vaccine dose [1–8]. However, important knowledge gaps remain regarding longitudinal changes in antibody levels before primary and booster vaccinations. For example, the timing of natural infection in relationship to vaccination and the resulting fluctuations in antibody levels over time may affect the magnitude of the immune response to subsequent vaccinations [9–13]. The degree to which immunity from natural SARS-CoV-2 infection influences the response to vaccination also remains a contested question [14–17]. The World Health Organization does not currently recommend prevaccination testing to guide vaccination policies [18]. Yet, it is possible that the optimal interdose interval may differ among individuals recently, remotely, and never infected [18–20]. This is of particular public health relevance in countries with limited access to SARS-CoV-2 vaccines [21], especially as many of them now have a high population seroprevalence from natural infection [22–25].

In addition, the modulating effect of prior SARS-CoV-2 infection on longitudinal changes in immune response to SARS-CoV-2 vaccination is yet to be definitively characterized. Multiple studies have demonstrated that adults infected with and/or vaccinated against SARS-CoV-2 sustain a detectable antibody response for a period of at least 6 months, but the longer-term durability and individual variability of the immune response remain less well described [26–30].

To explore these knowledge gaps, we examined longitudinal SARS-CoV-2 antibody responses among health care personnel (HCP) before and after vaccination and the association between natural infection and vaccination response.

## METHODS

### Patient Consent Statement

The study was approved by the University of North Carolina Institutional Review Board (20-0942), and all participants provided written informed consent.

### Study Design

We conducted a prospective observational cohort study of HCP at a large academic medical center as previously described [31]. HCP were considered eligible if they (1) provided patient care or support services at the medical center or the affiliated SARS-CoV-2 testing center; (2) planned to remain employed by the institution for the duration of the study; and (3) had access to stable internet, email, and a computer at home. Participants were enrolled between July 2020 and January 2021 and followed through February 2022. We collected blood samples for antibody measurements and midturbinate nasal swabs for SARS-CoV-2 testing by polymerase chain reaction (PCR) every 2 weeks for the first 12 weeks and monthly thereafter. Participants completed frequent electronic surveys as described previously [31]; they were specifically asked about any interval positive SARS-CoV-2 test result at enrollment, every 2 weeks for the first 36 weeks of participation, and monthly thereafter. Initial (V_1_) and second (V_2_) doses of mRNA-based SARS-CoV-2 ancestral monovalent vaccinations (Pfizer-BioNTech [BNT162b2] and Moderna [mRNA-1273]) were available to HCP through the occupational health clinic starting in mid-December 2020; booster doses (V_3_) were available starting October 2021.

### Antibody Testing

We measured total immunoglobulin (Ig) and immunoglobulin G (IgG) antibodies specific to the receptor-binding domain (RBD) of the SARS-CoV-2 spike protein in plasma by enzyme-linked immunosorbent assay (ELISA) as previously described [32]. We also measured RBD-specific immunoglobulin M (IgM) isotype levels for (1) participants with a documented SARS-CoV-2 infection prior to vaccination at all time points and (2) participants without evidence of infection prior to vaccination at the time point immediately prior to vaccination and at all post-V_1_ and post-V_2_ time points for which plasma sample was available. Finally, for all participants who had sufficient sample volume remaining (1466/1493, 98.2%), we measured IgG antibodies specific to the SARS-CoV-2 nucleocapsid protein at all time points. Each sample was run in duplicate. To account for plate-to-plate variability in optical density (OD), we calculated a positive-to-negative (P/N) ratio from the sample OD measurements as follows: average sample OD / average negative control OD (4 replicates) from the same plate [33–36]. The assay result was considered positive if the P/N value was above the following cutoffs: 2.57 for total Ig, 1.76 for IgM, and 2.40 for IgG, which were associated with >90% sensitivity and >98% specificity during validation [33] per the recommendations of the Centers for Disease Control and Prevention for SARS-CoV-2 serology assays [37].

### Live Virus Neutralization

We measured live virus neutralization using an ic-SARS-CoV-2 nanoluciferase neutralization assay [38] for the following participant samples: (1) the time point immediately prevaccination and 1 time point after each vaccination for seropositive cases with sufficient remaining volume and (2) a subset of samples from seronegative cases matched by sex, age, and time between vaccination and sample collection. Full-length D614G SARS-CoV-2 expressing nanoluciferase was designed and recovered by reverse genetics as previously described [39]. Vero C1008 cells (ATCC) were seeded the day prior to infection at 20 000 cells per well in black-bottom, black-walled 96-well plates (3916; Corning). Samples were tested at a starting dilution of 1:10 or 1:40. A positive-control human monoclonal antibody (S309; Adimab) was included on each plate at a starting concentration of 0.20 μg/mL. Samples were serially diluted 3-fold and then incubated with virus at 37 °C with 5% CO_2_ for 1 hour. After incubation, samples were added to cells in duplicate. Virus and cell controls and virus-only or background controls were included on each assay plate. Infection was allowed to proceed for 23 to 25 hours at 37 °C. After the incubation, the Nano-Glo Luciferase Assay System (N1130; Promega) was used according to the manufacturer's specifications to lyse cells and measure luciferase activity. The neutralization titer (EC50) was calculated as the dilution at which 50% reduction in relative light units was observed relative to the average of the virus and cell control wells subtracted by the average of background control wells.

### Definitions

Infection prior to vaccination was defined as a positive result from (1) a total Ig RBD-spike antibody ELISA test or (2) a SARS-CoV-2 PCR test performed as part of the study (between July 2020 and the date of first vaccination or last day of study participation, whichever came first) or as part of a clinical evaluation documented in the electronic medical record from January 2020 onward. Herein, we refer to participants with and without a SARS-CoV-2 infection prior to vaccination as “seropositive” and “seronegative,” respectively. Date of infection prior to vaccination was estimated from the first positive SARS-CoV-2 PCR test result, first positive total Ig RBD-spike antibody ELISA test result, or date of study entry for those who came into the study seropositive without a prior documented positive test result. Incident infection after 2 doses of an mRNA-based vaccine was defined as a positive SARS-CoV-2 PCR test result as documented in the electronic medical record, a positive PCR test result on study midturbinate nasal swab, any participant-reported positive test result in study surveys, or a change from a negative to positive result on nucleocapsid antibody testing for samples done as part of study testing. If the positive nucleocapsid result was an isolated result followed by negative results on 1 or more subsequent samples collected within 1 month, it was considered a false positive.

### Data Analysis

We plotted total Ig, IgG, and IgM antibody measurements by serostatus, conducted exact Wilcoxon rank sum tests, and calculated corresponding 95% CIs to compare P/N levels between seropositive and seronegative cases at the prevaccine, post-V_1_, post-V_2_, and post-V_3_ time points. Exact Wilcoxon signed rank tests were also conducted to evaluate change in P/N for those with paired pre- and postvaccination measurements. We fitted Loess curves to examine trends over time in total Ig P/N pre- and postvaccination, IgG P/N postvaccination, and total Ig P/N by number of days between vaccine dose and sample collection. Analyses were conducted with R version 4.2.2 (RStudio); neutralization curves were fit by Prism version 9 (GraphPad). No adjustments were made for multiple comparisons.

## RESULTS

### Study Cohort

We enrolled 213 HCP with 195 (92%) providing at least 1 blood sample. One was excluded due to coenrollment in a SARS-CoV-2 treatment trial. Two other participants were excluded because they enrolled after vaccination; thus, we were unable to ascertain their prevaccination serostatus. For the 192 individuals included, the median duration of follow-up was 4.5 months (IQR, 2.5–10.5), during which time the median number of samples provided per participant was 7 (range, 1–22). Twenty-eight participants (15%) contributed ≥15 samples (Supplementary Figure 1). The median age was 37 years (IQR, 32–44), 68% were female, and 66% identified as White and non-Hispanic (Table 1). The majority (189/192, 98%) received the BNT162b2 vaccine, and 99% (191/192) received at least 2 doses of a SARS-CoV-2 mRNA-based vaccine by the end of the study period. Paired antibody measurements before and after at least 1 vaccine dose were available for 99 participants, including 34 (34%) who had at least 1 measurement following a booster dose.

**Table 1.** Demographics, Serostatus, and Vaccinations for the Total Study Population and the Subsample of Participants With Pre- and Postvaccination Antibody Measurements

	Study Population, Median (IQR) or No. (%)		
Characteristic	Total (n = 192)	Pre- and Post-vaccine Antibody Results (n = 99)^a^	Post-booster Antibody Results (n = 34)
**Age, y**	37 (32–44)	38 (32–46)	40 (35–47)
**Female sex**	131 (68)	65 (66)	21 (62)
**Race/ethnicity**			
Non-Hispanic and Black	4 (2)	3 (3)	0 (0)
Non-Hispanic and Asian	9 (5)	4 (4)	2 (6)
Non-Hispanic and White	127 (66)	63 (64)	22 (64)
Non-Hispanic and other, ≥2 races, or unidentified	10 (5)	9 (9)	3 (9)
Hispanic ethnicity of any race	10 (5)	4 (4)	1 (3)
Other ethnicity of any race	12 (6)	4 (4)	4 (12)
Unidentified ethnicity of any race	5 (3)	3 (3)	1 (3)
Unidentified race and ethnicity	15 (8)	11 (9)	1 (3)
**Total Ig antibody serostatus before vaccination**			
Seronegative	182 (95)	93 (92)	30 (88)
Seropositive	10 (5)	6 (6)	4 (12)
At baseline visit	5 (3)	4 (4)	3 (9)
Occurred during follow-up	5 (3)	2 (2)	1 (3)
**Vaccinations received**			
None	1 (0.5)	NA	NA
2 doses	140 (73)	50 (51)	NA
3 doses	51 (27)	49 (49)	34 (100)

Abbreviations: Ig, immunoglobulin; NA, not applicable.

^a^Post-vaccine: after at least 1 dose.

### SARS-CoV-2 Infections

Ten participants (5%) were seropositive for SARS-CoV-2 prior to vaccination: 5 (3%) had antibodies at the baseline visit (prevalent seropositive), while 5 (3%) developed antibodies during the period of follow-up prior to vaccination (incident seropositive; Table 1). The median time from infection to first vaccination for seropositive cases was 3.0 months (IQR, 1.4–4.4). None required hospitalization. A total of 16 participants (8%) had an incident infection after receipt of any vaccination, 1 of which was a reinfection in a prevalent seropositive case. Pre- and postvaccine samples were available for 13 of these participants (81%), and postinfection samples were available for 7 (44%).

### Prevaccination Antibody Levels

**Figure 1.**
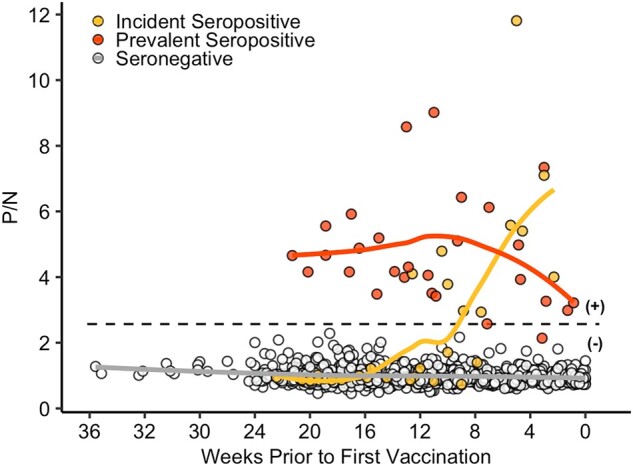
SARS-CoV-2–specific total immunoglobulin (Ig) antibody levels among health care personnel prior to vaccination. Total Ig positive-to-negative (P/N) ratios are shown over time in individuals who were SARS-CoV-2 seropositive at study entry (prevalent; red circles), became seropositive at some point during the study prior to vaccination (incident; yellow circles), or remained seronegative prior to vaccination (open circles). The x-axis represents weeks to first vaccine dose. The dotted line is a P/N ratio of 2.57, the cutoff associated with 99.3% specificity (SARS-CoV-2 Ig positive above the line, Ig negative below). Solid lines represent Loess curves for participants who were incident seropositive (yellow line), prevalent seropositive (red line), and seronegative (gray line).

**Figure 2.**
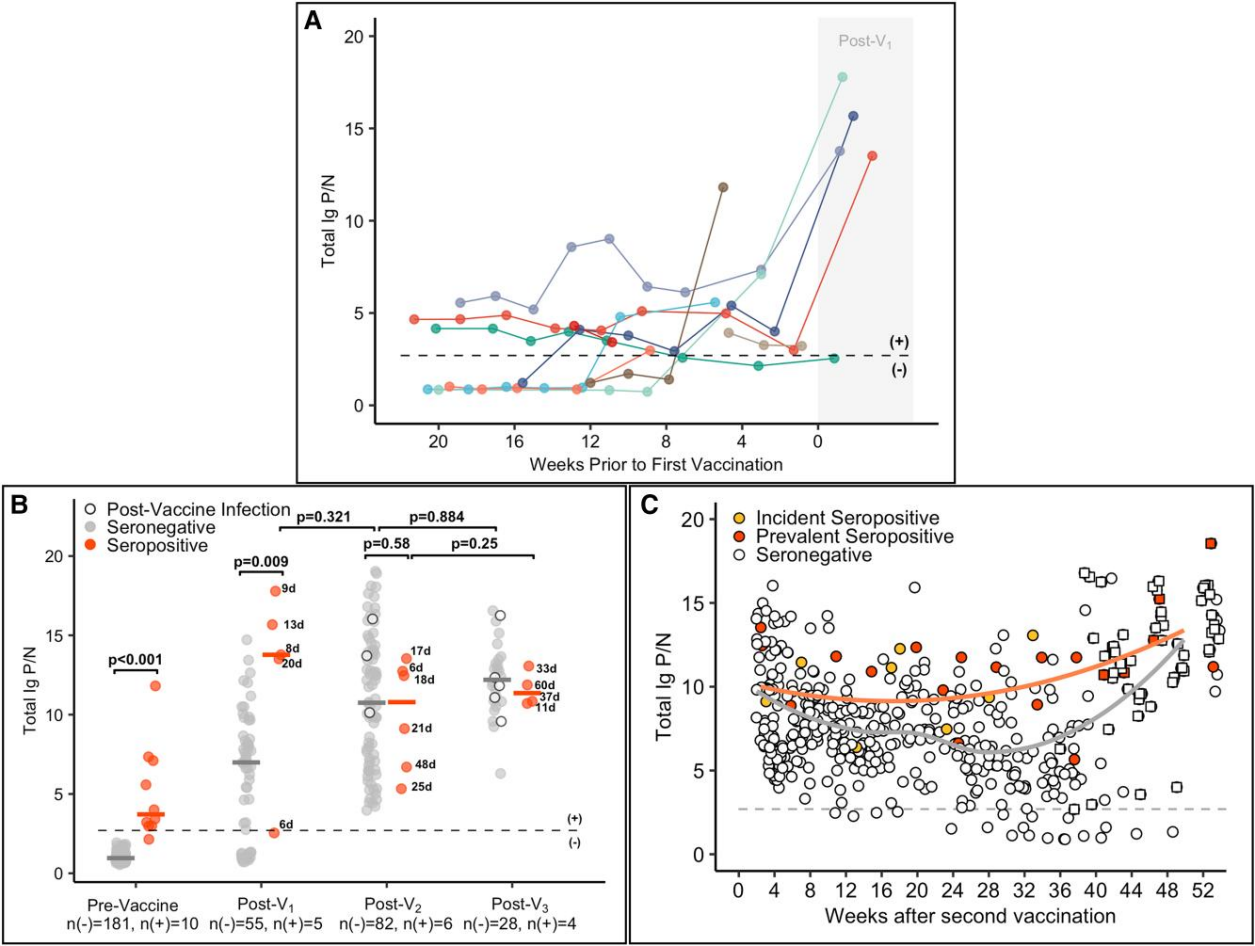
SARS-CoV-2–specific total immunoglobulin (Ig) antibody responses among health care personnel before and after vaccination against SARS-CoV-2 with an mRNA-based vaccine. *A*, Total Ig positive-to-negative (P/N) ratios over time in individuals who were SARS-CoV-2 seropositive at entry to the study (prevalent) or became seropositive at some point during the study prior to vaccination (incident). The dotted line is a P/N ratio of 2.57, the cutoff associated with 99.3% specificity (SARS-CoV-2 Ig positive above the line, Ig negative below). The x-axis represents weeks to first vaccine dose; values post–vaccine 1 (post-V_1_) are shaded. *B*, Total Ig P/N ratios at prevaccine, post-V_1_, post–vaccine 2 (post-V_2_), and post–booster dose (post-V_3_) time points by serostatus prior to vaccination: seronegative, n(–); seropositive, n(+). For the prevaccine time point, the most recent antibody level is shown prior to vaccination (for those who were vaccinated) or the most recent antibody level overall (for the 1 individual who was not vaccinated). For the postvaccine time points, the first measurement after 5 days postvaccination is included. The number of days between vaccination and sample collection for seropositive cases was as follows (median [range]): V_1_, 9 (6–20); V_2_, 20 (6–48); V_3_, 37 (11–60). For seronegative cases, the median time between V_1_ and sample collection was 16 days (range, 6–28); between V_2_ and sample collection, 20 days (6–77); and between V_3_ and sample collection, 39 days (6–106). The numbers next to seropositive circles indicate the number of days between vaccination and sample collection. Postinfection measurements for individuals who were infected with SARS-CoV-2 at any time after the first vaccine dose are shown as open circles. *C*, All total Ig P/N measurements after 5 days post-V_2_ for the entire study cohort: incident seropositive, yellow circles; prevalent seropositive, red circles; seronegative, open circles. Solid lines represent Loess curves for incident and prevalent seropositive cases combined (orange line) and those that were seronegative (gray line). P/N values from time points after the booster dose (post-V_3_) are shown as squares.

Prevaccination SARS-CoV-2–specific antibody responses are shown in Figures 1 and 2*A*. On average, total Ig antibody levels after natural infection were relatively low as compared with the responses to vaccination, although responses varied. Prior to vaccination, total Ig antibody levels decreased over time among prevalent seropositive cases, with 1 individual reverting to seronegative status approximately 17 weeks after presumed infection.

### Postvaccination Antibody Levels

We measured SARS-CoV-2–specific total Ig antibody responses to vaccination and compared the first measurement collected at least 5 days after the vaccine dose between seropositive and seronegative cases (Figure 2). Most participants who were seropositive (n = 4, 80%) exhibited a robust total Ig antibody response (2- to 5-fold increase in P/N) to their first vaccine with no temporal association between infection and vaccination, apart from the 1 participant who reverted to seronegative status just prior to vaccination (Figure 2*A*). Individuals who were seropositive at the time of initial vaccination (V_1_) developed an almost 2-fold higher antibody response than those who were seronegative (median P/N, 13.8 vs 7.0; difference estimate, 7.2 [95% CI, 1.8–12.2]; *P* = .009; Figure 2*B*). The post-V_1_ antibody response in seropositive cases was of similar magnitude to the post-V_2_ response among seronegative cases (post-V_1_ seropositive vs post-V_2_ seronegative, 13.8 vs 10.8; difference estimate, 2.8 [95% CI, −2.5 to 7.2]; *P* = .321). The absolute magnitude of the increase in antibody levels from pre- to post-V_1_ was also greater for seropositive than seronegative cases (median change in P/N from pre- to post-V_1_ seropositive vs seronegative, 10.5 vs 6.0; difference estimate, 3.2 [95% CI, −.8 to 6.6]; *P* = .130). The median total Ig for seronegative and seropositive cases was similar post-V_2_ (10.8 vs 10.8; difference estimate, −0.8 [95% CI, −4.6 to 2.6]; *P* = .576) and post-V_3_ (12.2 vs 11.4; difference estimate, −0.4 [95% CI, −2.6 to 1.3]; *P* = .564). The total Ig responses by days between vaccination and sample collection are shown in Supplementary Figure 2.

All post-V_2_ total Ig P/N measurements are shown in Figure 2*C*. An overall decline in antibody levels is noted after V_2_ that is more prominent for seronegative cases, but many of the seronegative and seropositive cases had P/N values above the positivity cutoff of 8 to 9 months after the initial vaccine series and prior to V_3_. Both groups showed an increase in antibody levels in response to V_3_ (squares; Figure 2*C*). The total Ig P/N trajectories for participants who contributed at least 15 samples are shown in Supplementary Figure 1.

**Figure 3.**
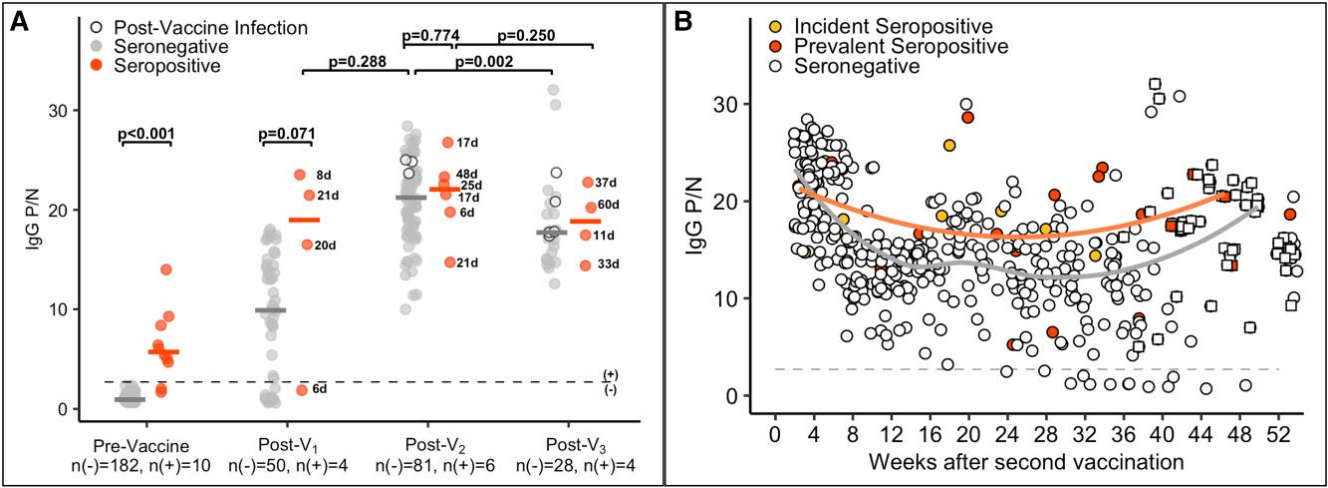
SARS-CoV-2–specific immunoglobulin G (IgG) subtype responses among health care personnel before and after vaccination against SARS-CoV-2 with an mRNA-based vaccine. *A*, SARS-CoV-2–specific IgG positive-to-negative (P/N) ratios at prevaccine, post–vaccine 1 (post-V_1_), post–vaccine 2 (post-V_2_), and postboost (post-V_3_) time points by serostatus prior to vaccination: seronegative, n(–); seropositive, n(+). For the prevaccine time point, the most recent antibody level is shown prior to vaccination (for those who were vaccinated) or the most recent antibody level overall (for the 1 individual who was not vaccinated). For the postvaccine time points, the first measurement after 5 days postvaccination dose is included. The dotted line is a P/N ratio of 2.40, the cutoff associated with 98.6% specificity (SARS-CoV-2 IgG positive above the line, IgG negative below). The numbers next to the circles indicate the number of days between vaccination and sample collection for seropositive cases. Postinfection measurements for individuals who were infected with SARS-CoV-2 at any time after the first vaccine dose are shown as open circles. *B*, All total IgG P/N measurements after 5 days post-V_2_ for the entire study cohort: incident seropositive, yellow circles; prevalent seropositive, red circles; seronegative, open circles. Solid lines represent Loess curves for incident and prevalent seropositive cases combined (orange line) and those that were seronegative (gray line). P/N values from time points after the booster dose (post-V_3_) are shown as squares.

We observed similar trends to what was seen with total Ig antibody levels when comparing IgG subtype responses with vaccination (Figure 3*A*). Notably, the median IgG P/N was higher post-V_1_ for seropositive as compared with seronegative cases (19.0 vs 9.9; difference estimate, 7.5 [95% CI, −.8 to 15.6]; *P* = .071) but similar between groups post-V_2_ (22.1 vs 21.2; difference estimate, 0.4 [95% CI, −3.1 to 3.9]; *P* = .774). When all post-V_2_ IgG measurements were evaluated (Figure 3*B*), there appeared to be a decline in antibody levels as time from initial vaccine series increased, although again most participants remained IgG positive 8 to 9 months after the initial 2-dose vaccine series. The IgG P/N trajectories for participants who contributed at least 15 samples are shown in Supplementary Figure 1.

IgM subtype responses are shown in Supplementary Figure 3. More than half (60%) of the seropositive cases were IgM negative at the nearest time point prior to vaccination. Postvaccine total Ig P/N ratios were similar between seropositive cases that were IgM positive and IgM negative prior to vaccination.

### Antibody Response and Infection

To further assess the association between SARS-CoV-2 infection and the magnitude and durability of the antibody response, we compared total Ig and IgG P/N measurements in aggregate between participants who were infected with SARS-CoV-2 at any time during the study and had at least 1 postinfection sample collected and those who remained uninfected throughout. For this analysis, participants with pre- and postvaccine samples were analyzed (n = 99), and pre- and postinfection measurements were included. We compared the measurements in 3 ways: all measurements prior to V_3_ (n = 99), post-V_2_ measurements, and post-V_3_ measurements (Table 2). For the first analysis, individuals who had an infection only after V_3_ were excluded. Participants who were infected had a higher median P/N than those who were uninfected for total Ig and IgG for all 3 analyses, with variability in the magnitude of the difference. The largest difference estimate was noted for post-V_2_ IgG, although with a wide confidence interval (infected vs uninfected, 16.72 vs 14.69; difference estimate, 6.52 [95% CI, 1.08–11.96]; *P* = .022).

**Table 2.** Anti-SARS-CoV-2 Total Ig and IgG P/N Levels Between Participants Infected During Follow-up With at Least 1 Postinfection Sample and Those Never Infected

	All Pre-V_3_ Measurements				Post-V_2_ Measurements Only				Post-V_3_ Measurements Only			
	Median	DE	95% CI	*P* Value	Median	DE	95% CI	*P* Value	Median	DE	95% CI	*P* Value
Total Ig												
Infected	5.10	1.63	.89–2.37	<.001	8.84	4.83	1.86–7.81	.003	12.34	1.40	.60–2.21	<.001
Uninfected	1.31				7.29				12.07			
IgG												
Infected	6.81	2.14	.73–3.55	.003	16.72	6.52	1.08–11.96	.022	18.65	1.69	.33–3.07	.016
Uninfected	1.37				14.69				15.71			

Analysis was limited to participants for whom at least 1 prevaccination and 1 postvaccination sample was collected (n = 99) and included pre- and postinfection measurements. For the analysis of all pre-V_3_ measurements, individuals who had only a post-V_3_ infection were excluded.

Abbreviations: DE, difference estimate; Ig, immunoglobulin; IgG, immunoglobulin G; P/N, positive-to-negative ratio; post-V_1_, post–vaccine 1; post-V_2_, post–vaccine 2; post-V_3_, postboost.

### Live Virus Neutralization

**Figure 4.**
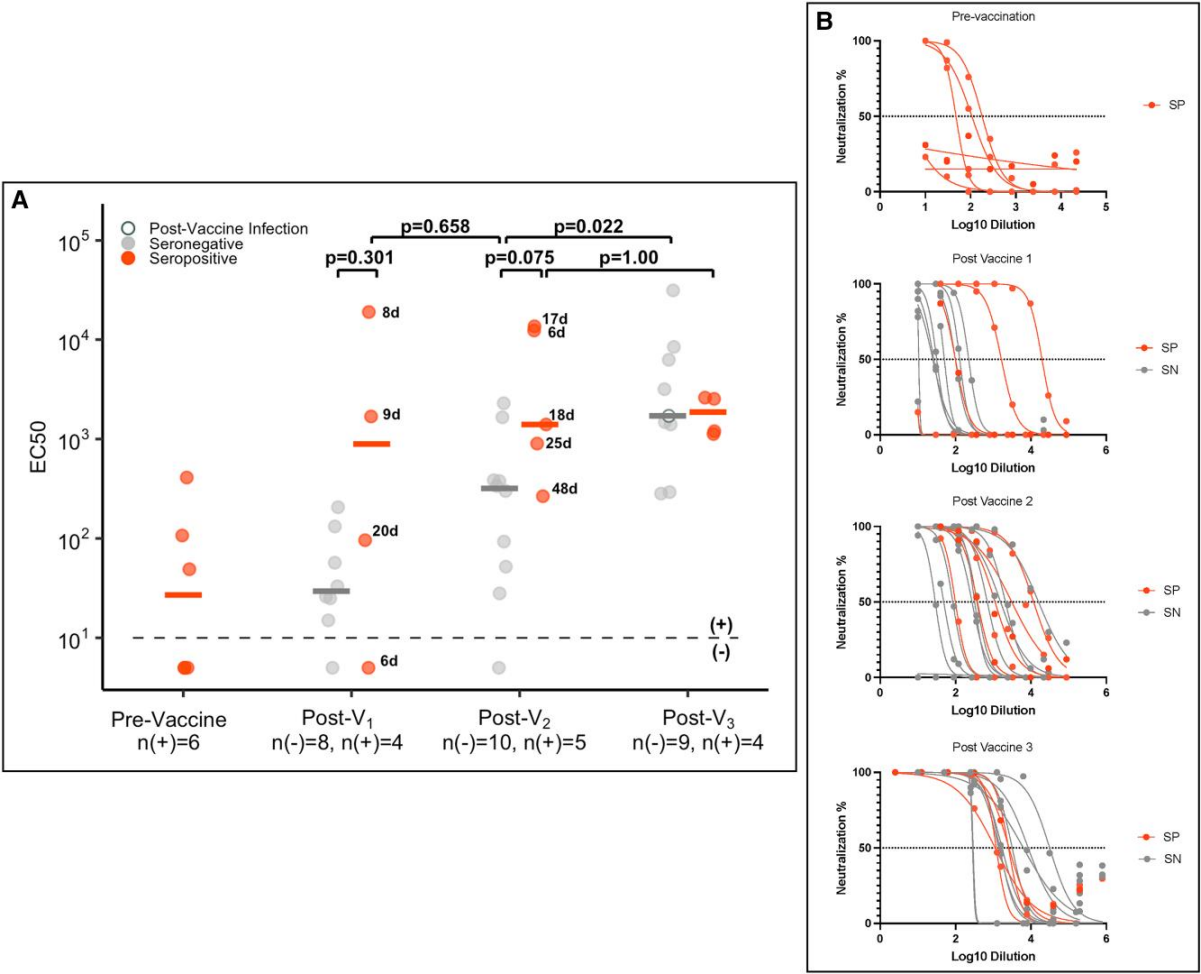
SARS-CoV-2 D614G live virus neutralization among health care personnel by serostatus prior to vaccination. *A*, EC50 of the SARS-CoV-2 D614G live virus neutralization titers prevaccine, post–vaccine 1 (post-V_1_), post–vaccine 2 (post-V_2_), and postboost (post-V_3_) for all seropositive cases (red) with remaining sera and a subset of seronegative cases (gray). Samples for seronegative cases were selected by matching age and time between vaccination and sample collection to the samples from seropositive cases, as described in the Methods section. Black numbers next to the circles indicate the number of days between vaccination and sample collection for seropositive cases. Postinfection measurements for individuals who were infected with SARS-CoV-2 at any time after the first vaccine dose are shown as open circles. *B*, Corresponding neutralization curves. SN, seronegative; SP, seropositive.

We measured live virus neutralization for a subset of participants prevaccine (n = 6), post-V_1_ (n = 12), post-V_2_ (n = 15), and post-V_3_ (n = 13; Figure 4*A* and 4*B*). Prior to vaccination, 3 seropositive cases (50%) did not have detectable neutralizing antibodies despite a history of natural infection. We observed wide variability in post-V_1_ neutralization titers among those who were seropositive and seronegative, but all participants who were seropositive developed neutralization titers ≥10^2^ post-V_2_, which was not true of all participants who were seronegative. There was an absolute difference in the post-V_1_ and post-V_2_ median log_10_ EC50 between seropositive and seronegative cases, but corresponding confidence intervals were wide and included zero (post-V_1_, 2.6 vs 1.5; log_10_ difference estimate, 1.0 [95% CI, −.7 to 2.8]; *P* = .301; post-V_2_, 3.1 vs 2.5; log_10_ difference estimate, 1.0 [95% CI, −.1 to 2.2]; *P* = .075). There was an increase in median log_10_ EC50 for participants who were seronegative between post-V_2_ and post-V_3_ (2.5 vs 3.2; log_10_ difference estimate, 1.2 [95% CI, .4–1.8]; *P* = .022) that was not observed among participants who were seropositive (3.1 vs 3.2; log_10_ difference estimate, 0.01 [95% CI, −1.1 to 1.0]; *P* > .99).

One individual reverted from seropositive to seronegative prior to vaccination (dark orange line, Figure 2*A*) and had a relatively lower antibody response to the first vaccine dose. In a sensitivity analysis in which this participant was removed, there was a difference in neutralization titer between seropositive and seronegative cases at 2 time points: post-V_1_ (difference logEC50, 1.76 [95% CI, .2–2.9]; *P* = .048) and post-V_2_ (difference logEC50, 1.47 [95% CI, .4–2.4]; *P* = .024; data not shown).

## DISCUSSION

We report longitudinal antibody responses before and after SARS-CoV-2 vaccination in a cohort of HCP early in the COVID-19 pandemic. We observed several interesting and important findings. First, prior SARS-CoV-2 infection was associated with a greater magnitude of antibody response to the first dose of mRNA-based SARS-CoV-2 vaccine, even if the infection was asymptomatic or caused only mild disease. Second, while we did not note any difference in the total Ig or IgG response immediately after V_2_ or V_3_ among previously seropositive vs seronegative cases, neutralization did appear higher after V_2_ among those who were seropositive; both findings have been observed in prior studies [7, 40–42]. Third, when antibody responses throughout study follow-up were analyzed in aggregate, infection at any point during the study was associated with an increased magnitude of antibody levels in this vaccinated cohort. Finally, the antibody response to the initial 2-dose vaccine series waned over time but persisted above the positivity cutoff for the assay for 8 to 9 months, and the magnitude appeared to be more stable over time in seropositive cases vs seronegative ones. A booster dose then restored the antibody response to levels seen immediately post-V_2_ in both groups.

Our results corroborate previous studies that noted higher peak antibody titers [1–5, 7, 8], neutralization titers [1, 2, 4], and T-cell responses [2] after the first dose of an mRNA-based vaccine in those with a history of SARS-CoV-2 infection as compared with those who had not been previously infected. We extend previous work by including multiple prevaccine time points from seropositive cases, demonstrating that their robust antibody response to the first dose of vaccine seems to be consistent regardless of the their varied patterns of anti-spike and neutralizing antibody responses before vaccination, including those who exhibited low antibody levels nearing the assay cutoff just prior to vaccination. Furthermore, a robust antibody response occurred in individuals who were seropositive regardless of the interval between infection and vaccination (Figure 2*A*), a finding that is consistent with prior work [11, 12, 42]. Interestingly, these and other studies found that more remote infections (>5 months prior to vaccination) are associated with a higher magnitude of the antibody response to vaccination as compared with infections occurring closer to the time of the first vaccine dose [11–13]. The participants who were seropositive in our study were infected at a median 3 months prior to vaccination; the small number of seropositive cases in our study limits our ability to compare shorter and longer intervals. Regardless, our results support that past infection regardless of timing provides a benefit to the primary vaccination immune response [41, 43].

We found that the total Ig and IgG antibody levels in response to natural infection prior to vaccination are quantitatively lower than in response to vaccination. In fact, some of our cohort did not develop neutralizing antibody titers in response to natural infection, consistent with a prior study in North Carolina [33]. This important finding underscores the importance of vaccination for those who have been infected.

While it is known that vaccine effectiveness against infection wanes with time from completion of the primary vaccine series [26, 44–46], there are few studies describing the long-term durability of antibody responses to vaccination, with and without prior natural infection [14, 40]. Our findings provide important information about the timing and resilience of the immune response, demonstrating that anti-spike SARS-CoV-2 antibody levels wane but appear to persist for most people at least 8 to 9 months and up to a year after completion of the initial vaccine series, regardless of serostatus prior to vaccination. This is consistent with studies measuring antibody responses with less frequent sampling and for shorter intervals postvaccination (up to 6 months) [26–28, 40, 45]. We observed a trend toward a more stable magnitude of antibody level over time postvaccination in seropositive cases as compared with seronegative ones prior to vaccination. This is in contrast to the conclusions of a previous study from a similar cohort of HCP in the United States that noted a similar trajectory over time between those with severe COVID-19 infection prior to vaccination and those without evidence of infection [14]. However, the investigators did observe significant differences at several time points and when comparing individuals vaccinated with BNT162b2 at 180 to 300 days following vaccination, as was the case for our study cohort. While our sample size of participants infected prior to vaccination is not large enough to draw definitive conclusions on antibody persistence, overall our findings appear consistent with prior data [14, 28, 40].

A small cohort remained in the study at the time of the first booster dose, allowing us to evaluate the kinetics of its SARS-CoV-2–specific antibody response from prevaccine to post-V_3_ for up to 18 months of total follow-up. We noted an increase in total Ig and IgG antibody response after the booster dose to levels similar to what was seen immediately post-V_2_ when looking at all values in aggregate (Figures 2*B* and 3*B*). This was consistent with previous studies demonstrating enhanced antibody response and immune protection against SARS-CoV-2 infection following a third dose [47–49]. While we did note a decrease in IgG but not total Ig among seronegative cases when comparing participants’ first measurements after V_3_, this could be explained by a difference in days between vaccination and sample collection (post-V_3_ vs post-V_2_, 38.5 vs 20). We did not observe a difference in antibody response or neutralization after V_3_ between prevaccine seropositive and seronegative cases. However, we did observe a small difference in the post-V3 antibody response when comparing those infected at any time during study follow-up and those who were never infected. Furthermore, we likely underestimated this association because of the inclusion of preinfection data points in the analysis. This finding is consistent with other studies that observed how hybrid immunity a with booster dose provides the best protection against symptomatic infection by the Omicron BA.1 variant [20, 50, 51].

Our study had notable strengths, including the frequency and duration of sampling (up to 5 months before vaccination and 13 months after the initial vaccine series) and the total number of antibody measurements. Yet, our study is limited by a small sample size of participants who were seropositive and those who acquired infection postvaccination, which likely reflects high levels of adherence to use of personal protective equipment and physical distancing among a generally healthy, hospital-based cohort of HCP. Despite this limitation, our results are consistent with the findings of previous studies, and we detected a significant difference in the total Ig antibody response to the first vaccination between seropositive and seronegative cases using an exact nonparametric statistical test. Most samples in this analysis were collected prior to the emergence of the B.1.617.2 (Delta) and BA.1 (Omicron) variants. Follow-up continued and postvaccination infections were noted through both these surges, although our analysis is limited by the smaller number of participants who remained in the study at that time. Finally, we do not report cellular immunity over time, which is likely a key component of vaccine effectiveness and may be modified by a history of natural infection [52, 53].

In conclusion, SARS-CoV-2 infection, even when months prior to vaccination, appears to “prime” the immune response to the first dose of mRNA-based vaccine, although its modulatory effect on subsequent doses is less clear. In addition, our findings support earlier research showing that antibody response to the primary vaccination series against SARS-CoV-2 wanes but persists for long periods. Finally, although our study is limited by its sample size and therefore underpowered, our results suggest that infection at any time before or after vaccination may be associated with higher antibody levels over time as compared with full vaccination but without infection. Together, our results suggest that knowledge of prior infection is important in understanding the robustness and durability of immunity from mRNA vaccines. Future research should examine whether memory T-cell responses are modified by prior infection and how infection after vaccination affects future booster responses.

## Supplementary Material

ofae009_Supplementary_Data

## Supplementary Data

Supplementary materials are available at *Open Forum Infectious Diseases* online. Consisting of data provided by the authors to benefit the reader, the posted materials are not copyedited and are the sole responsibility of the authors, so questions or comments should be addressed to the corresponding author.

## Notes

***Acknowledgments.*** We thank the participants for their willingness to contribute to advancing our understanding of SARS-CoV-2 and its impact on HCP, especially during the early and uncertain months of the pandemic. We also appreciate the hard work of the graduate students from the University of North Carolina Gillings School of Global Public Health (Elle Law, MPH, Elyse Miller, MPH, and Paul Zivich, MPH, PhD) and the phlebotomists who contributed to the sample collection, protocols, and day-to-day implementation of the study (these individuals received compensation for their work on the study). We thank Subhashini Sellers, MD, MSc, and Meghan Rebuli, PhD (University of North Carolina School of Medicine), and Bailey Fosdick, PhD (Colorado State University Department of Statistics), for their support of this study and its analysis and Ralph Baric, PhD (University of North Carolina Gillings School of Global Public Health), for use of his biosafety level 3 laboratory and reagents for the neutralization assays. These contributors did not receive any compensation for their role in the study.

***Author contribution.*** E. J. C. designed and supervised the study, composed and revised the article, and helped to secure study funding. D. R. Z. contributed to study design, led the data analysis, and assisted with data and figure generation. S. H. assisted with data analysis and visualization and revised the article. R. A. assisted with data collection and analysis and revised the article. A. K. G. assisted with data collection and analysis and revised the article. E. K. L. contributed to study design, assisted with generation of the figures, and revised the article. B. E. S.-S. supervised the data analysis and revised the article. H. A., H. E. G., and E. K. generated and analyzed the data and assisted with revision of the article. N. A. and R. R. assisted with study design and implementation. J. L. T. assisted with study design and implementation. C. B. assisted with study design and implementation and revised the article. C. C. assisted with study design and implementation. C. E. M. assisted with study implementation and data analysis and revised the article. D. J. W. assisted with study design and revised the article. A. J. M. and L. P. assisted with data analysis and interpretation and revised the article. J. J. J. assisted with study design and supervised data collection in the laboratory. R. M. B. conceptualized, designed, and supervised the study; revised the article; and helped to secure study funding. A. E. A. conceptualized, designed, and supervised the study; composed and revised the article; and helped to secure study funding. All authors participated in preparing the manuscript and have reviewed and approved its final submitted version.

***Presentation history.*** The results reported in this article were presented in part at the IDWeek 2022 conference in a hybrid poster/oral presentation format as part of the COVID-19 Vaccines Rapid Fire Poster Session in the IDWeek Hot Zone (22 October 2022). Preliminary analyses were also published in preprint form on medrxiv.org: Ciccone EJ, Zhu DR, Ajeen R, et al. SARS-CoV-2 seropositivity after infection and antibody response to mRNA-based vaccination. medRxiv. **2021**. https://doi.org/10.1101/2021.02.09.21251319.

***Financial support.*** This work was supported by a 2007 Gillings Gift from the UNC Gillings School of Global Public Health to A. E. A; the North Carolina Policy Collaboratory at the University of North Carolina at Chapel Hill with funding from the North Carolina Coronavirus Relief Fund established and appropriated by the North Carolina General Assembly to R. M. B.; the UNC School of Medicine to R. M. B.; a generous donation to the UNC School of Medicine by an anonymous donor to R. M. B.; the National Institute for Occupational Safety and Health (75D30121P10086 0000HCCK-2021 to A. E. A. and E. J. C.); the National Science Foundation (NSF 20-4353 to A. E. A.); the National Institutes of Health (R01 EB025021 to A. E. A.); National Center for Advancing Translational Sciences, National Institutes of Health (UL1TR001111), for use of the REDCap database; the National Heart, Lung, and Blood Institute (5T32HL007106 to E. J. C.); the National Institute of Allergy and Infectious Diseases (NIAID; 5F30AI160898 to D. R. Z.); the National Institute of Environmental Health Sciences (T32ES007018 to S. H.; F30ES032302 to E. K. L.); the NIAID (5K23AI141764 to R. M. B.; 5K24AI134990 to J. J. J.); the University of North Carolina at Chapel Hill Center for AIDS Research, an National Institutes of Health–funded program (P30AI050410 to B.E.S.-S.); and the National Cancer Institute (1U54CA260543 to L. P.) to produce and supply the necessary antigens for the study.
