# Supplementary figures and images for: Magnitude and Durability of the Antibody Response to mRNA-Based Vaccination Among SARS-CoV-2 Seronegative and Seropositive Health Care Personnel
Source: Open Forum Infect Dis. 2024 Jan 19;11(1):ofae009. doi: 10.1093/ofid/ofae009 (PMC10826795; doi:10.1093/ofid/ofae009)

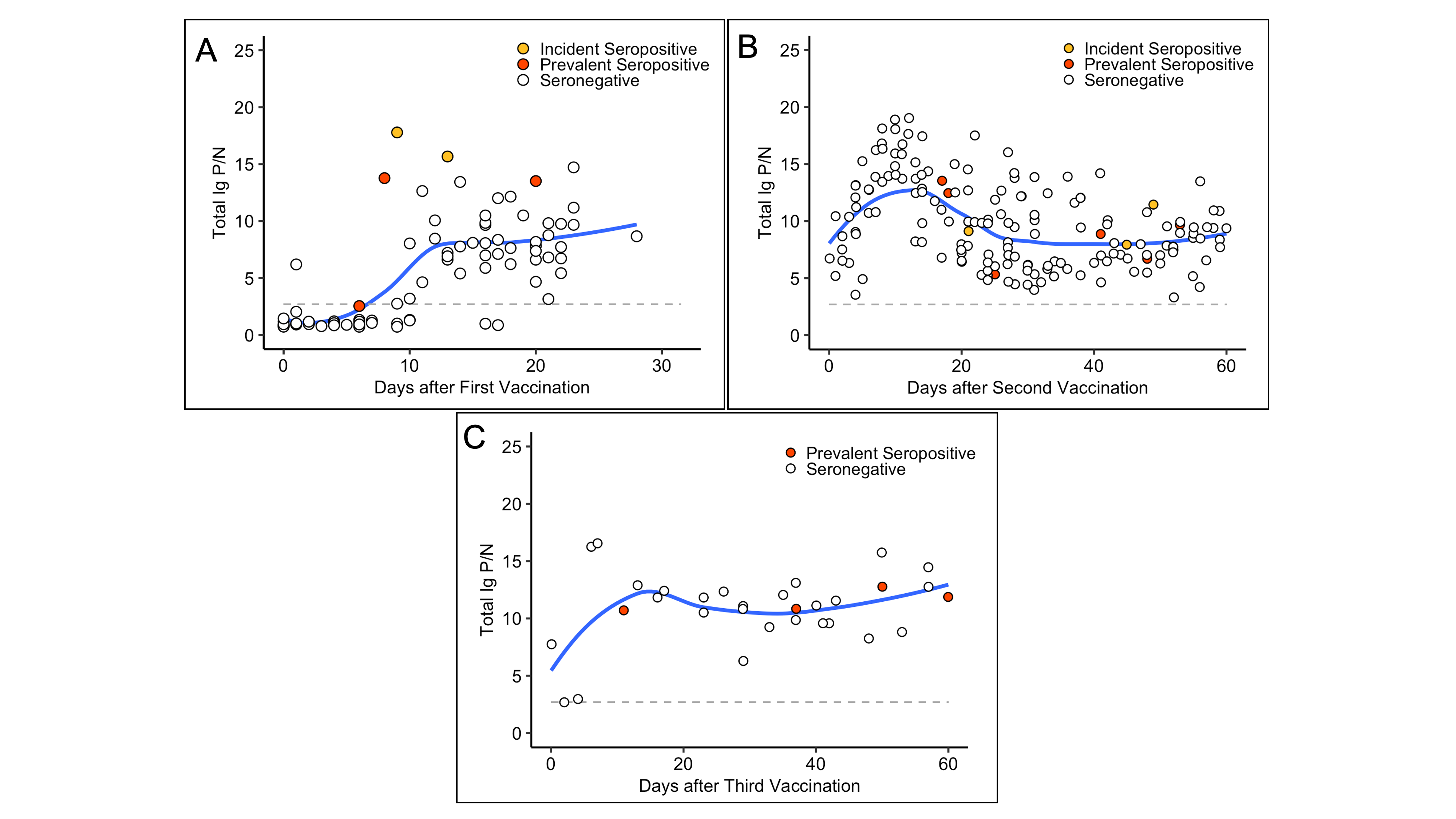

Supplement: ofae009_Supplementary_Data [file ofae009_supplementary_data.zip › FigS2.tiff]

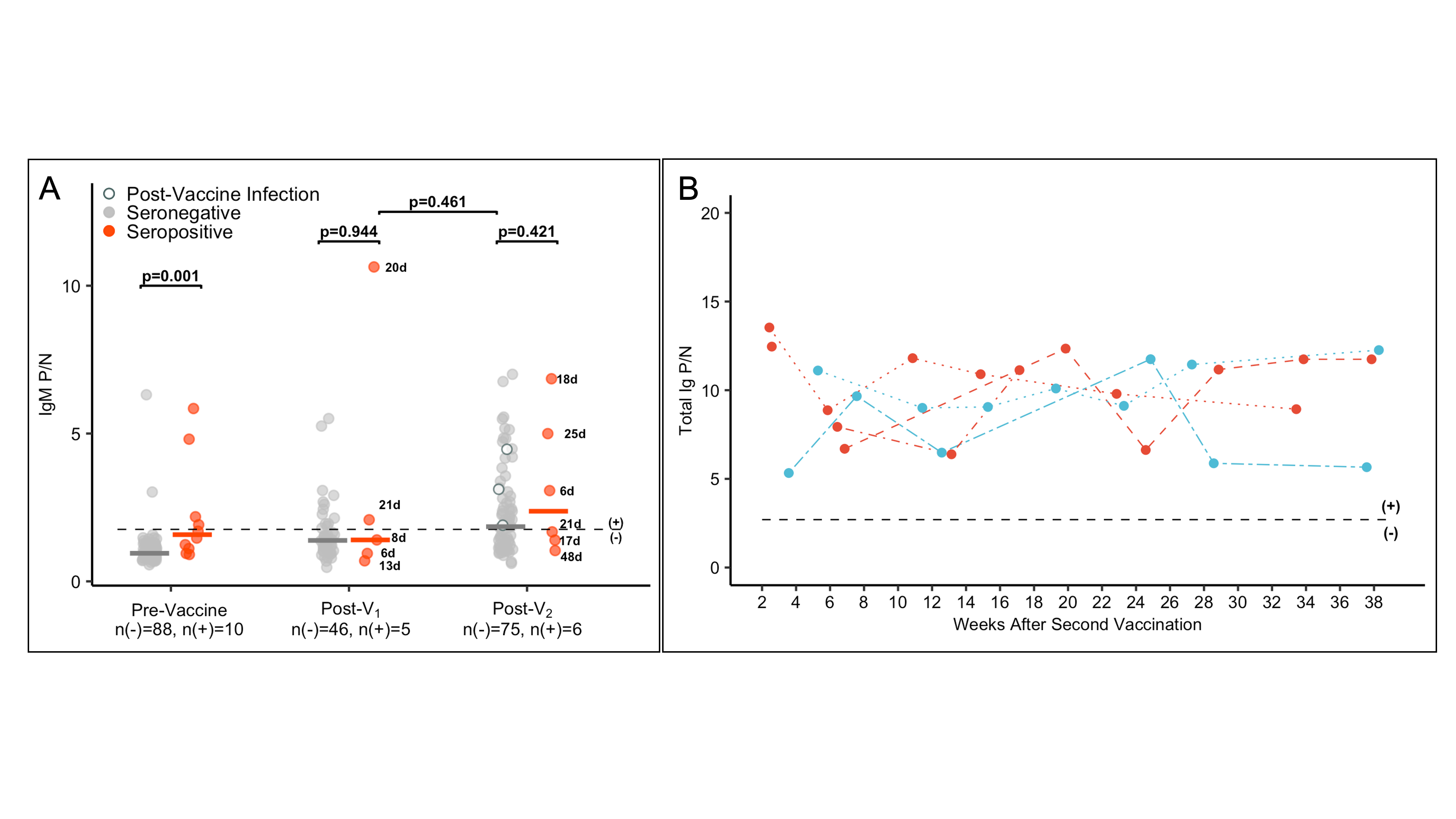

Supplement: ofae009_Supplementary_Data [file ofae009_supplementary_data.zip › FigS3.tiff]

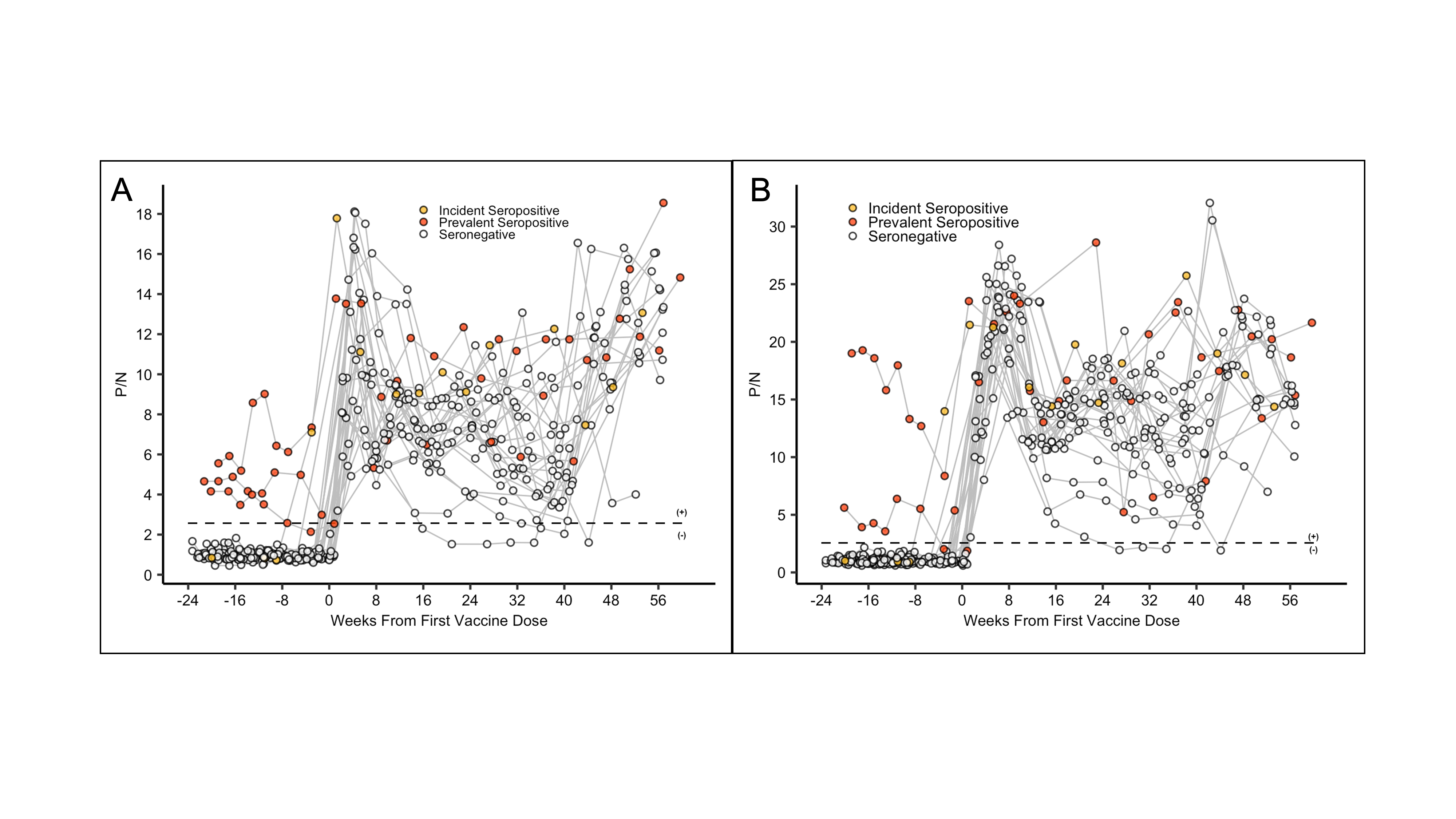

Supplement: ofae009_Supplementary_Data [file ofae009_supplementary_data.zip › FigS1.tiff]
